# Quality-of-Life Evaluation of Patients with Unresectable Locally Advanced or Locally Recurrent Head and Neck Carcinoma Treated with Head and Neck Photoimmunotherapy

**DOI:** 10.3390/cancers14184413

**Published:** 2022-09-11

**Authors:** Isaku Okamoto, Takuro Okada, Kunihiko Tokashiki, Kiyoaki Tsukahara

**Affiliations:** Department of Otorhinolaryngology, Head and Neck Surgery, Tokyo Medical University, 6-7-1 Nishishinjuku, Shinjuku-ku, Tokyo 160-0023, Japan

**Keywords:** head and neck photoimmunotherapy, cetuximab sarotalocan sodium, unresectable locally advanced or locally recurrent head and neck carcinoma, quality of life

## Abstract

**Simple Summary:**

Head and neck photoimmunotherapy (HN-PIT) is a new treatment developed for local control of head and neck carcinoma. This study assessed the quality of life (QOL) of nine patients with unresectable locally advanced or locally recurrent head and neck carcinoma (LA/LR-HNC) treated with HN-PIT. QOL was compared before and 4 weeks after HN-PIT. There were no significant changes in all the QOL assessment parameters after treatment with HN-PIT. For patients with unresectable LA/LR-HNC, HN-PIT provided good local control without decreasing the QOL.

**Abstract:**

Head and neck photoimmunotherapy (HN-PIT), a new treatment developed for local control of head and neck carcinoma, uses cetuximab sarotalocan sodium with a laser system to specifically destroy only tumor cells. No studies have examined the impact of HN-PIT on the quality of life (QOL) of patients with head and neck cancer. This study assessed the QOL of patients with unresectable locally advanced or locally recurrent head and neck carcinoma (LA/LR-HNC) treated with HN-PIT. Nine eligible patients with unresectable LA/LR-HNC who underwent HN-PIT at our institution between 20 January 2021 and 30 April 2022 were included in the study. They completed a QOL evaluation form. The primary endpoint was QOL assessment. The secondary endpoints were overall response rate, overall survival (OS), progression-free survival, and adverse events. QOL was compared before and 4 weeks after HN-PIT. There were no significant changes in all QOL assessment parameters after treatment with HN-PIT. The overall response rate was 89%, and safety was acceptable. For patients with unresectable LA/LR-HNC, HN-PIT provided good local control without decreasing the QOL. The addition of HN-PIT to conventional head and neck carcinoma treatment may lead to the prolongation of OS in head and neck carcinoma.

## 1. Introduction

The head and neck region contains important organs involved in activities such as mastication, swallowing, breathing, and speech, which affect the quality of life (QOL) of individuals. Local control of head and neck cancer in these areas contributes not only to prolonging survival but also to the maintenance of QOL. In addition, there is a significant impact on cosmetic outcomes. According to the National Comprehensive Cancer Network (NCCN) guidelines, the standard treatment for locally advanced recurrent metastatic squamous cell carcinoma of the head and neck is primarily pharmacotherapy [1]. Category 1 immune checkpoint inhibitors nivolumab and pembrolizumab are highly therapeutic and used primarily in first-line therapy [2,3]. Furthermore, immune checkpoint inhibitors are associated with fewer adverse events than conventional chemotherapy and are less likely to cause a decline in the QOL [2,4]. However, pharmacotherapy is aimed at disease control and is not a curative treatment. If tumor progression becomes difficult to control, the best supportive care is to be provided.

Head and neck photoimmunotherapy (HN-PIT) is a new treatment developed for local control of head and neck carcinoma [5,6]. HN-PIT uses cetuximab sarotalocan sodium in combination with a laser system. Cetuximab sarotalocan sodium is formed by an antibody-photosensitive complex that combines cetuximab, an epidermal growth factor receptor monoclonal antibody, with the dye IR700, a photosensitive substance. After administration of cetuximab sarotalocan sodium, only tumor cells are specifically destroyed by illumination with a red light at 690 nm. According to a report summarizing the results of a phase I/IIa multicenter, open-label study of HN-PIT, the overall response rate (ORR) was 43.3% (95% confidence interval (CI) 25.46–62.57%) for treatment efficacy. The median overall survival (OS) was 9.30 months (95% CI 5.16–16.92), and the median progression-free survival (PFS) was 5.16 months (95% CI 2.10–5.52) [7]. In Japan, cetuximab sarotalocan sodium received manufacturing and marketing approval in September 2020. Since January 2021, HN-PIT could be selected as a treatment strategy for unresectable locally advanced or locally recurrent head and neck carcinoma (LA/LR-HNC) [8]. Generally, patients with unresectable LA/LR-HNC who are candidates for HN-PIT have previously undergone free-flap surgery for head and neck carcinoma. However, free-flap surgery for head and neck carcinoma patients tends to result in a reduced QOL. Moreover, salvage reconstructive surgery after free-flap surgery may result in a significantly reduced QOL.

To the best of our knowledge, there are no studies including real-world data on the impact of HN-PIT on the QOL of patients with head and neck carcinoma. At our institution, patients with head and neck carcinoma who have recurrent or metastatic disease are asked to complete a QOL assessment questionnaire to understand changes in their QOL during treatment. We also routinely assess patients’ QOL using HN-PIT as an indicator for the continuation or modification of treatment. Therefore, we conducted a retrospective study of the HN-PIT assessment of QOL. The purpose of this study was to assess the QOL of patients with unresectable LA/LR-HNC who underwent HN-PIT. We did not find a significant change in the QOL of patients with LA/LR-HNC after HN-PIT; thus, HN-PIT provided good local control without decreasing the QOL.

## 2. Materials and Methods

### 2.1. Study Design

This was a single-center, retrospective study conducted at Tokyo Medical University Hospital in Japan to evaluate the QOL of patients with unresectable LA/LR-HNC treated with HN-PIT. The study was approved by the institutional review board of the Center for Research Administration and Innovation, Tokyo Medical University (T2022-0022; 20 May 2022). The study complied with the tenets of the Declaration of Helsinki, and written consent to participate in the study was obtained from all patients.

### 2.2. Patients

Patients with unresectable LA/LR-HNC who underwent HN-PIT at Tokyo Medical University Hospital between 20 January 2021, and 30 April 2022 and who completed the QOL assessment form were eligible. Patients with hypersensitivity to any component of cetuximab sarotalocan sodium, patients with tumor invasion into the carotid artery, and patients who could be treated with standard therapy such as chemoradiation were excluded from HN-PIT. Patients who refused to participate in the study were excluded from the study.

### 2.3. Outcomes and Assessments

The primary endpoint was the QOL assessment. The QOL was assessed using the European Organization for Research and Treatment of Cancer (EORTC) Quality of Life Questionnaire (QLQ) Core 30 Module (QLQ-C30) [9], a basic QOL questionnaire used for patients with malignancies, and the EORTC QLQ Head and Neck Cancer Module (QLQ-H&N35) [10], a disease-specific questionnaire. Patients answered both the EORTC QLQ-C30 and QLQ-H&N35 questionnaires. QOL was assessed by comparing the scores at two time points: before and 4 weeks after HN-PIT. HN-PIT can be performed for up to four cycles with a minimum interval of 4 weeks between cycles. Therefore, we decided to assess the QOL at 4 weeks post treatment. These scores ranged from 0 to 100, with higher scores indicating higher functioning and symptom burden. The EORTC QLQ-C30 version 3.0 was used for scoring. However, for the global health status parameters, higher scores indicated lower functioning and symptom burden. The secondary endpoints were ORR, OS, PFS, and adverse events. The period of OS was defined as the duration between the date of cetuximab sarotalocan sodium initiation and the date of the last follow-up or the patient’s death, whichever occurred first. The period of PFS was defined as the duration between the date of cetuximab sarotalocan sodium initiation and the date of objective disease progression or the patient’s death from any cause, whichever occurred first. Tumor response was assessed by two radiologists at our institution according to the Response Evaluation Criteria in Solid Tumors Guideline (version 1.1) [11]. Tumor-node-metastasis classification was determined according to the Union for International Cancer Control, version 7, criteria [12]. The adverse events were assessed using the common terminology criteria for adverse events, version 4.0. [13].

### 2.4. Drug Administration of Cetuximab Sarotalocan Sodium

Cetuximab sarotalocan sodium is a light-sensitive substance complex that must be administered under light shielding. The intravenous bag was covered with a light-shielding cover, and in-line filters and tubing were covered with aluminum foil to protect them from light. Cetuximab sarotalocan sodium (640 mg/m^2^) was administered intravenously over 2 h. To prevent photosensitivity, the brightness in the room was set to no more than 120 lx. Since laser irradiation is performed 20–28 h after the end of cetuximab sarotalocan sodium administration, the start time of the surgery was adjusted such that laser irradiation could be performed within this timeframe.

### 2.5. Statistical Analysis

A repeated-measures linear mixed model was applied with each QOL score as the dependent variable, time as a fixed factor, subjects as a variable factor, and the repeated-measures covariance structure as compound symmetry. The least square mean and its 95% CI at each measurement point were calculated. The estimated mean and 95% CI for the change from before HN-PIT were also calculated in the same way, and a significance test of the change relative to before HN-PIT was performed. No correction for multiplicity was made. *p* < 0.05 indicated a significant difference. OS and PFS were estimated using the Kaplan–Meier method. Statistical analysis was performed using EZR [14] and SPSS statistics version 22.0 (IBM Japan, Ltd., Tokyo, Japan).

## 3. Results

### 3.1. Characteristics of the Patients

Ten patients underwent HN-PIT between 20 January 2021 and 30 April 2022. The median follow-up time was 179 (57–479) days. All patients completed the QOL evaluation form; however, one patient was excluded because he had not undergone HN-PIT for 4 weeks during the study period. Thus, nine patients were included in the study. Table 1 shows the clinical characteristics of the patients. Target lesions extending to multiple subsites in a single patient were counted separately. The primary sites were the oropharynx in two patients, oral cavity in three, hypopharynx in two, and larynx in two. For the oropharynx, the sites of the target lesions for HN-PIT were the anterior wall in five patients, lateral wall in three, posterior wall in two, and superior wall in one (Table 2). Target lesions in the oral cavity subregion occurred in the buccal mucosa in one patient, the upper gingiva in one patient, the lower gingiva in one patient, and the tongue in one patient. Other regions where lesions occurred were reconstructed skin valve sites in two patients, cervical lymph node in one patient, and maxillary sinus in one patient. Regarding the treatment history, previous surgery and previous radiation therapy were performed in nine patients (100%) and previous chemotherapy in two patients (22%). The reasons for unresectability included difficulty in reconstructive surgery due to technique or patient preference in seven patients and iatrogenic multiple-recurrent lesions after radiotherapy or multiple surgeries in two patients.

### 3.2. QOL Assessment

**Figure 1:**
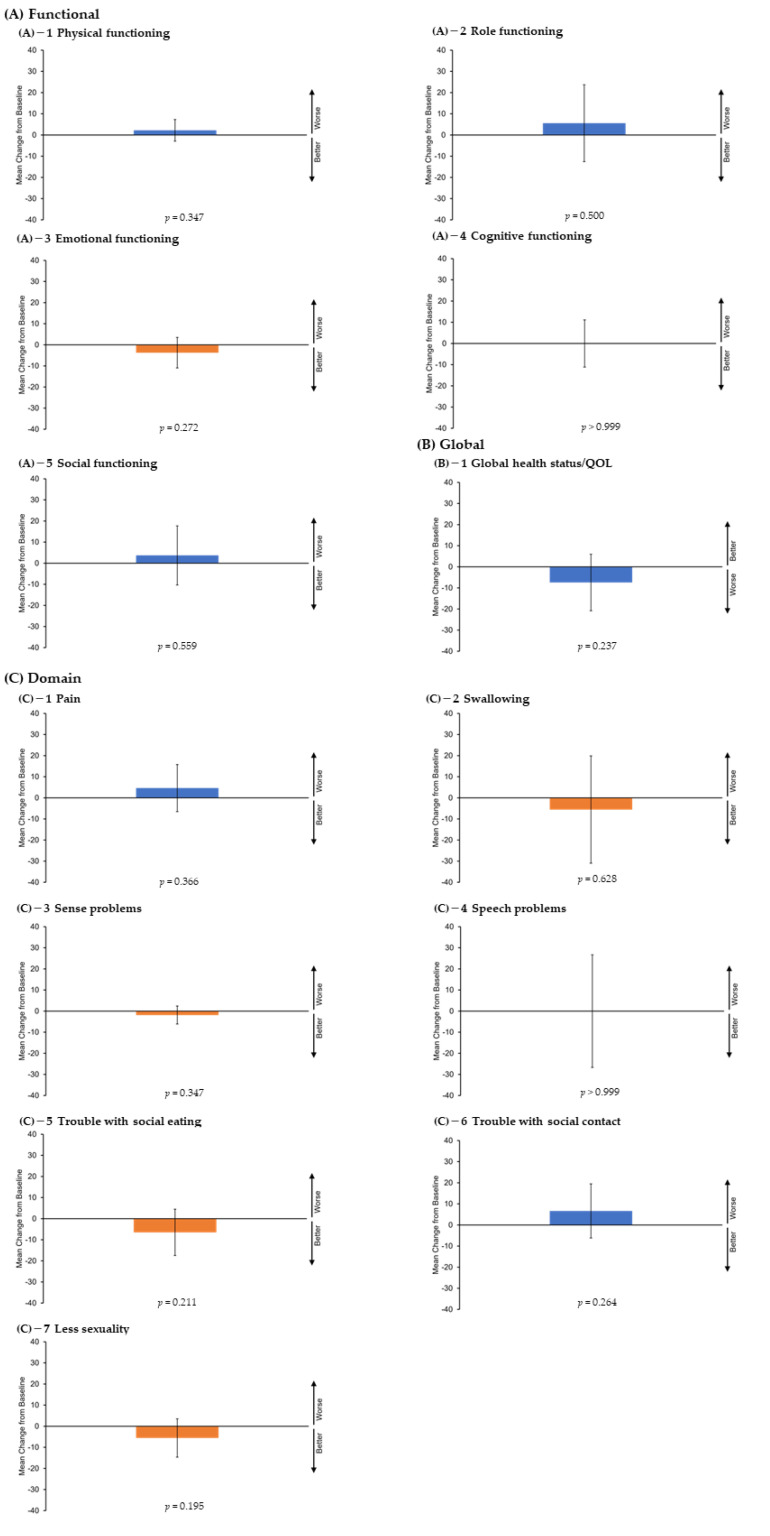
Quality of life assessments. (**A**) Functional scales (physical, role, emotional, cognitive, and social activities) and (**B**) global health status were assessed using the EORTC QLQ-C30. (**C**) Domain scales (pain, swallowing, sense problems, speech problems, trouble with social eating, trouble with social contact, and reduced sexuality) were assessed using the QLQ-H&N35. All scales ranged from 0 to 100, and score changes of at least 10 points were considered clinically significant. Higher values for functional and domain scales indicate poor functioning, whereas higher values for global health status indicate better functioning. The I bar indicates 95% confidence intervals. QOL, quality of life; EORTC QLQ-C30, European Organization for Research and Treatment of Cancer Quality of Life Questionnaire Core 30 Module; QLQ-H&N35, Quality of Life Questionnaire Head and Neck Cancer Module.

Functional scales (physical, role, emotional, cognitive, and social functioning) and global health status were assessed using the EORTC QLQ-C30. Domain scales (pain, swallowing, sense problems, speech problems, trouble with social eating, trouble with social contact, and reduced sexuality) were assessed using the QLQ-H&N35. Table 3 and Figure 1 show the results of the QOL assessment after 4 weeks of HN-PIT. There was no significant change in any of the QOL assessment parameters after HN-PIT.

### 3.3. Efficacy

**Figure 2:**
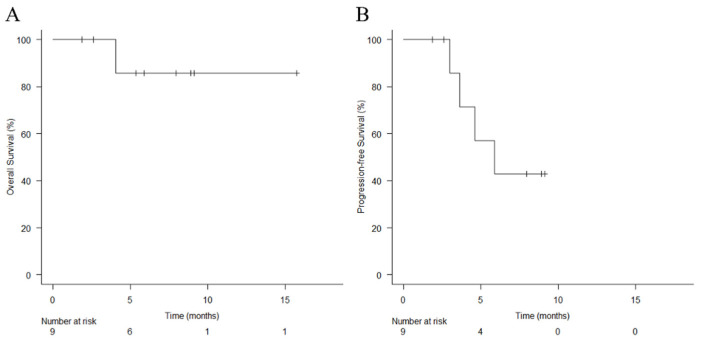
Kaplan–Meier curves of (**A**) overall survival and (**B**) progression-free survival. Vertical lines show censored events.

The responses to treatment were as follows: complete response (two patients), partial response (six patients), and stable disease (one patient). The ORR was 89%, and the disease-control rate (DCR) was 100%. Four patients had progression after HN-PIT: two had distant metastases, and two had perineural carotid artery extension. All four patients were treated with pembrolizumab, but one patient did not respond and was shifted to the best supportive care and died. The median OS was not calculable (N/C) (95% CI, 4.0 months to N/C), and the 1-year OS rate was 85.7% (95% CI, 33.4–97.9%) (Figure 2A). The median PFS was N/C (95% CI, 5.9 months to N/C), and the 1-year PFS rate was N/C (Figure 2B).

### 3.4. Safety

Table 4 lists the adverse events in all patients who underwent HN-PIT. Mucositis occurred in eight patients (89%). Edema of the larynx was observed in three patients (33%) who had not undergone laryngectomy, and edema of the pharynx was observed in patients who had undergone laryngectomy. No patient required emergency tracheostomy. Hemorrhage occurred in two patients (22%), but it was from the needle puncture site, and the bleeding stopped within a few minutes. Acneiform rash, a cetuximab-specific adverse event, occurred in one patient (11%). In one patient (11%), the tip of the needle catheter broke during HN-PIT and remained in the tumor.

## 4. Discussion

This study aimed to assess the QOL of patients with unresectable LA/LR-HNC who underwent HN-PIT by comparing QOL scores at two time points: before and 4 weeks after HN-PIT. The results of the study showed no significant decrease or improvement in any of the QOL endpoints with HN-PIT. HN-PIT can be performed for up to four cycles with a minimum interval of 4 weeks between cycles. In addition, HN-PIT is subject to acute adverse events due to treatment for approximately 2 weeks after the start date of treatment, which stabilize after 4 weeks. Therefore, the optimal time to assess QOL was determined to be 4 weeks after treatment. The “swallowing and trouble with social eating” QOL item scores tended to improve. This may be because patients who had difficulty eating owing to recurrent lesions in the pharyngeal space were able to eat more easily after HN-PIT as the obstruction was improved. However, the pain remained after 4 weeks of HN-PIT. A higher dose may be needed for pain control. However, the QOL assessment parameters did not change significantly after HN-PIT. We interpreted the results of this study as indicating that HN-PIT is not a treatment that causes a marked decrease in QOL.

The secondary endpoints were ORR, OS, PFS, and adverse events. As for the efficacy, the ORR was 89%, and the DCR was 100%. A phase I/IIa multicenter, open-label study on HN-PIT reported the recommended dose, safety, and therapeutic efficacy [7,15]. The primary objective of the phase I part was to determine the recommended dose of RM-1929 and optimal laser light intensity under fixed-light intensity. A total of nine patients, three at each dose, were enrolled, and the recommended drug dose was determined to be 640 mg/m^2^; the optimal laser light intensity was 50 J/cm^2^ for superficial lesions and 100 J/cm for deep lesions. In the phase IIa part, 30 patients received RM-1929 photoimmunotherapy to confirm its safety and therapeutic efficacy. Treated patients received a median of 2 cycles (1–4 cycles) for a total of 65 cycles of photoimmunotherapy. Grade 3 or higher adverse events included anemia, dysphagia, oral pain, pneumonia, laser site pain, local edema, hyponatremia, tumor hemorrhage, and tumor pain. Thirteen (43.3%) patients reported serious adverse events, and three cases were considered to be treatment-related. In terms of the treatment response, the ORR was 43.3% (95% CI 25.46–62.57%), complete response was achieved in 4 (13.3%) patients, partial response in 9 (30.0%) patients, and disease control in 24 (80.0%) patients (95% CI 61.43–92.29%). The median OS was 9.30 months (95% CI 5.16–16.92), and the median PFS was 5.16 months (95% CI 2.10–5.52). A comparison between the treatment effects of HN-PIT and immune checkpoint inhibitors and other pharmacologic therapies should be considered. In the CheckMate 141 study [2,16], the median OS for nivolumab was 7.7 months (95% CI 5.7--8.8), with a 2-year OS rate of 16.9% (95% CI 12.4–22.0); the median PFS was 2.1 months (95% CI 2.0–3.4), and the ORR was 13.3%. Real-world studies of nivolumab in Japanese patients with head and neck cancer showed a median OS of 6.3–13.4 months, a median PFS of 2.5–6.5 months, and an ORR of 15.0–46.2% [17,18,19,20,21,22,23,24,25,26,27]. The results of the current study compared favorably with pharmacotherapy with immune checkpoint inhibitors. In the current study, the median values for OS and PFS were not reached due to the short observation period. The efficacy of HN-PIT needs to be reevaluated after an extended observation period. Further studies are needed to make comparisons with HN-PIT and pharmacotherapy.

In terms of safety, it is necessary to separately consider HN-PIT-related adverse events and adverse events from previous medical conditions; pain and mucositis were the most common adverse events related to HN-PIT. Patients with laryngeal preservation tended to develop edema at the treated site. These patients should be carefully managed for laryngeal edema, including opting for prophylactic tracheostomy. As for photosensitivity, our institution has taken measures such as careful dark room management [28], and no photosensitivity was found. As a complication of surgery, it is important to be careful not to break the tip of the needle catheter. Needle catheter tips are very soft and can be easily broken if they strike bone or other surfaces. In this study, the needle catheter tip broke in one patient (11%) and remained in the tumor. This is thought to have occurred when the needle catheter was inserted through the cervical approach into a tumor on the anterior wall of the mid-pharynx and broke when it hit the hyoid bone. Hemodialysis was initiated during the observation period in one patient with acute kidney injury. This patient had chronic renal failure due to diabetes and had been considered for hemodialysis before HN-PIT. His renal function gradually worsened, and dialysis was initiated after HN-PIT was completed.

HN-PIT is considered a treatment for unresectable LA/LR-HNC with no reduction in QOL and good local control. The safety was also acceptable. The NCCN guidelines recommend nivolumab [2] and pembrolizumab [3] for the treatment of unresectable head and neck carcinoma. In Japan, HN-PIT is now an option in addition to these drugs. However, whether HN-PIT should be used as a first-line treatment is controversial. We consider that HN-PIT should be given priority over pharmacotherapy for four reasons: first, pharmacotherapy is not a curative treatment. Second, HN-PIT is very effective for local control. Third, HN-PIT does not decrease the QOL. Fourth, when HN-PIT is ineffective, pharmacotherapeutic options exist. HN-PIT causes necrosis of cancer cells, which release damage-associated molecular patterns (DAMPs) such as ATP, calreticulin, and high mobility group box 1 (HMGB1). Cancer antigens and DAMPs activate the immune system, leading to immunogenic cell death (ICD). ICD promotes dendritic cell maturation and antigen presentation to CD8-positive T cells, thereby inducing tumor immunity [6]. Furthermore, activation of the immune system may also have an effect on cancer cells at non-irradiated sites and cancer cell lesions at distant metastatic sites [29], which is known as the abscopal effect [30]. Although we have not experienced the abscopal effect, detailed elucidation of the tumor microenvironment after HN-PIT is desirable for a more effective HN-PIT.

The limitations of the studies are as follows. To date, there have been no real-world studies of HN-PIT excluding clinical trials and case reports, and no comparison can be made with other studies. In addition, only nine patients were analyzed in this study, and the non-inferiority of HN-PIT in terms of QOL could not be proven. However, considering the limited number of target patients, it is desirable to actively report the results from various perspectives. Large-scale multicenter studies on the therapeutic efficacy of HN-PIT are desirable and are being conducted mainly at facilities specializing in head and neck carcinoma in Japan. Most importantly, HN-PIT is now an option for the treatment of conventional head and neck carcinoma. The HN-PIT option should be used aggressively when there are indications for treatment. This may ultimately lead to the prolongation of OS in patients with head and neck carcinoma.

## 5. Conclusions

HN-PIT is the only treatment approved by the insurance system for head and neck carcinoma in Japan. This study assessed the QOL of patients with unresectable LA/LR-HNC who underwent HN-PIT. For these patients, HN-PIT did not decrease QOL and had a good local control rate. The safety was also acceptable. HN-PIT may prolong OS in head and neck carcinoma.

## Figures and Tables

**Table 1:** Clinical characteristics.

Clinical Characteristics	All Patients (*n* = 9)	
	No.	%
**Age, years**		
Mean	73	
Median	73	
Range	67–77	
**Sex**		
Male	8	89
Female	1	11
**ECOG performance status**		
PS 0	9	100
PS 1	0	0
**Primary tumor site**		
Oropharynx	2	22
p16-positive	0	-
p16-negative	2	-
Oral cavity	3	33
Hypopharynx	2	22
Larynx	2	22
**T category**		
T0	1	11
T1	1	11
T2	3	33
T3	2	22
T4	2	22
**N category**		
N0	8	89
N1/N2/N3	1	11
**M category**		
M0	9	100
M1	0	0
**Previous surgery**		
Yes	9	100
No	0	0
**Previous radiation therapy**		
Yes	9	100
No	0	0
**Previous chemotherapy**		
Yes	2	22
No	7	78
**Reason for unresectability**		
Difficulty in reconstructive surgery	7	78
Iatrogenic multiple recurrent lesions after radiotherapy or multiple surgeries	2	22

ECOG, Eastern Cooperative Oncology Group; PS, performance status.

**Table 2:** Location of target lesion.

Case	Primary Tumor Site	Location of Target Lesion
1	Larynx	Cervical lymph node
2	Oral cavity	Buccal mucosa
3	Hypopharynx	Oropharynx (anterior wall/lateral wall/superior wall)
4	Oropharynx	Oropharynx (anterior wall)
5	Oral cavity	Tongue/upper gingiva/lower gingiva
6	Oral cavity	Maxillary sinus
7	Larynx	Reconstructed skin valve site/oropharynx (anterior wall/posterior wall)
8	Oropharynx	Reconstructed skin valve site/oropharynx (anterior wall/lateral wall)
9	Hypopharynx	Oropharynx (anterior wall/lateral wall/posterior wall)

**Table 3:** Changes in quality-of-life scores.

	QOL Score LS Mean (95% CI)	Change from Baseline LS Mean (95% CI)	*p*-Value vs. Baseline
**Functional scales**			
Physical functioning			
Pre-HN-PIT	88.1 (80.7–95.6)	-	-
4 weeks	90.4 (83.0–97.8)	2.2 (−2.9–7.3)	0.347
Role functioning			
Pre-HN-PIT	85.2 (70.9–99.4)	-	-
4 weeks	90.7 (76.5–105.0)	5.6 (−12.6–23.7)	0.500
Emotional functioning			
Pre-HN-PIT	89.8 (80.4–99.2)	-	-
4 weeks	86.1 (76.7–95.5)	−3.7 (−10.9–3.5)	0.272
Cognitive functioning			
Pre-HN-PIT	87.0 (73.7–100.4)	-	-
4 weeks	87.0 (73.7–100.4)	0.0 (−11.1–11.1)	>0.999
Social functioning			
Pre-HN-PIT	88.9 (77.7–100.1)	-	-
4 weeks	92.6 (81.4–103.8)	3.7 (−10.3–17.7)	0.559
**Global health status**			
Global health status/QoL			
Pre-HN-PIT	68.5 (51.7–85.3)	-	-
4 weeks	61.1 (44.3–77.9)	−7.4 (−20.8–6.0)	0.237
**Domain scales**			
Pain			
Pre-HN-PIT	21.3 (5.1–37.5)	-	-
4 weeks	25.9 (9.8–42.1)	4.6 (−6.5–15.8)	0.366
Swallowing			
Pre-HN-PIT	30.6 (10.7–50.5)	-	-
4 weeks	25.0 (5.1–44.9)	−5.6 (−31.0–19.9)	0.628
Sense problems			
Pre-HN-PIT	9.3 (−7.7–26.2)	-	-
4 weeks	7.4 (−9.6–24.4)	−1.9 (−6.1–2.4)	0.347
Speech problems			
Pre-HN-PIT	34.6 (18.8–50.3)	-	-
4 weeks	34.6 (18.8–50.3)	0.0 (−26.7–26.7)	>0.999
Trouble with social eating			
Pre-HN-PIT	33.3 (14.2–52.4)	-	-
4 weeks	26.9 (7.8–46.0)	−6.5 (−17.5–4.5)	0.211
Trouble with social contact			
Pre-HN-PIT	9.6 (−4.0–23.3)	-	-
4 weeks	16.3 (2.6–29.9)	6.7 (−6.1–19.5)	0.264
Less sexuality			
Pre-HN-PIT	18.5 (−6.5–43.6)	-	-
4 weeks	13.0 (−12.1–38.0)	−5.6 (−14.6–3.5)	0.195

QOL, quality of life; LS mean, least square mean; 95% CI, 95% confidence interval; HN-PIT, head and neck photoimmunotherapy.

**Table 4:** Adverse events.

Patients, *n* (%)	Grade	Grade	Grade	Grade	Grade	Grades
	1	2	3	4	5	all
Pain	1 (11)	7 (78)	1 (11)	0 (0)	0 (0)	9 (100)
Mucositis	1 (11)	6 (68)	1 (11)	0 (0)	0 (0)	8 (89)
Laryngeal edema	0 (0)	2 (22)	0 (0)	1 (11)	0 (0)	3 (33)
Dysphagia	0 (0)	1 (11)	2 (22)	0 (0)	0 (0)	3 (33)
Tongue and pharyngeal edema	0 (0)	2 (22)	1 (11)	0 (0)	0 (0)	3 (33)
Nausea	3 (33)	0 (0)	0 (0)	0 (0)	0 (0)	3 (33)
Hyponatremia	0 (0)	0 (0)	2 (22)	0 (0)	0 (0)	2 (22)
Hemorrhage	2 (22)	0 (0)	0 (0)	0 (0)	0 (0)	2 (22)
Diarrhea	2 (22)	0 (0)	0 (0)	0 (0)	0 (0)	2 (22)
Acute kidney injury	0 (0)	0 (0)	0 (0)	1 (11)	0 (0)	1 (11)
Anemia	0 (0)	0 (0)	1 (11)	0 (0)	0 (0)	1 (11)
Hypokalemia	0 (0)	0 (0)	1 (11)	0 (0)	0 (0)	1 (11)
Liver dysfunction	0 (0)	0 (0)	1 (11)	0 (0)	0 (0)	1 (11)
Weight loss	0 (0)	0 (0)	1 (11)	0 (0)	0 (0)	1 (11)
Acneiform rash	0 (0)	1 (11)	0 (0)	0 (0)	0 (0)	1 (11)
Fever	0 (0)	1 (11)	0 (0)	0 (0)	0 (0)	1 (11)
Aspiration	0 (0)	1 (11)	0 (0)	0 (0)	0 (0)	1 (11)
Hyperkalemia	0 (0)	1 (11)	0 (0)	0 (0)	0 (0)	1 (11)
Trismus	0 (0)	1 (11)	0 (0)	0 (0)	0 (0)	1 (11)
Constipation	0 (0)	1 (11)	0 (0)	0 (0)	0 (0)	1 (11)
Dehydration	0 (0)	1 (11)	0 (0)	0 (0)	0 (0)	1 (11)
Intratumoral broken needle fragments	0 (0)	1 (11)	0 (0)	0 (0)	0 (0)	1 (11)
Oral dysesthesia	1 (11)	0 (0)	0 (0)	0 (0)	0 (0)	1 (11)
Edema of face	1 (11)	0 (0)	0 (0)	0 (0)	0 (0)	1 (11)

## Data Availability

Not applicable.
